# Clustering identifies endotypes of traumatic brain injury in an intensive care cohort: a CENTER-TBI study

**DOI:** 10.1186/s13054-022-04079-w

**Published:** 2022-07-27

**Authors:** Cecilia A. I. Åkerlund, Anders Holst, Nino Stocchetti, Ewout W. Steyerberg, David K. Menon, Ari Ercole, David W. Nelson, the CENTER-TBI Participants and Investigators, Cecilia Åkerlund, Krisztina Amrein, Nada Andelic, Lasse Andreassen, Audny Anke, Anna Antoni, Gérard Audibert, Philippe Azouvi, Maria Luisa Azzolini, Ronald Bartels, Pál Barzó, Romuald Beauvais, Ronny Beer, Bo-Michael Bellander, Antonio Belli, Habib Benali, Maurizio Berardino, Luigi Beretta, Morten Blaabjerg, Peter Bragge, Alexandra Brazinova, Vibeke Brinck, Joanne Brooker, Camilla Brorsson, Andras Buki, Monika Bullinger, Manuel Cabeleira, Alessio Caccioppola, Emiliana Calappi, Maria Rosa Calvi, Peter Cameron, Guillermo Carbayo Lozano, Marco Carbonara, Simona Cavallo, Giorgio Chevallard, Arturo Chieregato, Giuseppe Citerio, Hans Clusmann, Mark Coburn, Jonathan Coles, Jamie D. Cooper, Marta Correia, Amra Čović, Nicola Curry, Endre Czeiter, Marek Czosnyka, Claire DahyotFizelier, Paul Dark, Helen Dawes, Véronique De Keyser, Vincent Degos, Francesco Della Corte, Hugo den Boogert, Bart Depreitere, Đula Đilvesi, Abhishek Dixit, Emma Donoghue, Jens Dreier, GuyLoup Dulière, Ari Ercole, Patrick Esser, Erzsébet Ezer, Martin Fabricius, Valery L. Feigin, Kelly Foks, Shirin Frisvold, Alex Furmanov, Pablo Gagliardo, Damien Galanaud, Dashiell Gantner, Guoyi Gao, Pradeep George, Alexandre Ghuysen, Lelde Giga, Ben Glocker, Jagoš Golubovic, Pedro A. Gomez, Johannes Gratz, Benjamin Gravesteijn, Francesca Grossi, Russell L. Gruen, Deepak Gupta, Juanita A. Haagsma, Iain Haitsma, Raimund Helbok, Eirik Helseth, Lindsay Horton, Jilske Huijben, Peter J. Hutchinson, Bram Jacobs, Stefan Jankowski, Mike Jarrett, Jiyao Jiang, Faye Johnson, Kelly Jones, Mladen Karan, Angelos G. Kolias, Erwin Kompanje, Daniel Kondziella, Evgenios Kornaropoulos, LarsOwe Koskinen, Noémi Kovács, Ana Kowark, Alfonso Lagares, Linda Lanyon, Steven Laureys, Fiona Lecky, Didier Ledoux, Rolf Lefering, Valerie Legrand, Aurelie Lejeune, Leon Levi, Roger Lightfoot, Hester Lingsma, Andrew I. R. Maas, Ana M. CastañoLeón, Marc Maegele, Marek Majdan, Alex Manara, Geoffrey Manley, Costanza Martino, Hugues Maréchal, Julia Mattern, Catherine McMahon, Béla Melegh, David Menon, Tomas Menovsky, Ana Mikolic, Benoit Misset, Visakh Muraleedharan, Lynnette Murray, Ancuta Negru, David Nelson, Virginia Newcombe, Daan Nieboer, József Nyirádi, Otesile Olubukola, Matej Oresic, Fabrizio Ortolano, Aarno Palotie, Paul M. Parizel, JeanFrançois Payen, Natascha Perera, Vincent Perlbarg, Paolo Persona, Wilco Peul, Anna Piippo-Karjalainen, Matti Pirinen, Dana Pisica, Horia Ples, Suzanne Polinder, Inigo Pomposo, Jussi P. Posti, Louis Puybasset, Andreea Radoi, Arminas Ragauskas, Rahul Raj, Malinka Rambadagalla, Isabel Retel Helmrich, Jonathan Rhodes, Sylvia Richardson, Sophie Richter, Samuli Ripatti, Saulius Rocka, Cecilie Roe, Olav Roise, Jonathan Rosand, Jeffrey V. Rosenfeld, Christina Rosenlund, Guy Rosenthal, Rolf Rossaint, Sandra Rossi, Daniel Rueckert, Martin Rusnák, Juan Sahuquillo, Oliver Sakowitz, Renan SanchezPorras, Janos Sandor, Nadine Schäfer, Silke Schmidt, Herbert Schoechl, Guus Schoonman, Rico Frederik Schou, Elisabeth Schwendenwein, Charlie Sewalt, Ranjit D. Singh, Toril Skandsen, Peter Smielewski, Abayomi Sorinola, Emmanuel Stamatakis, Simon Stanworth, Robert Stevens, William Stewart, Ewout W. Steyerberg, Nino Stocchetti, Nina Sundström, Riikka Takala, Viktória Tamás, Tomas Tamosuitis, Mark Steven Taylor, Braden Te Ao, Olli Tenovuo, Alice Theadom, Matt Thomas, Dick Tibboel, Marjolein Timmers, Christos Tolias, Tony Trapani, Cristina Maria Tudora, Andreas Unterberg, Peter Vajkoczy, Shirley Vallance, Egils Valeinis, Zoltán Vámos, Mathieu van der Jagt, Gregory Van der Steen, Joukje van der Naalt, Jeroen T. J. M. van Dijck, Inge A. van Erp, Thomas A. van Essen, Wim Van Hecke, Caroline van Heugten, Dominique Van Praag, Ernest van Veen, Thijs Vande Vyvere, Roel P. J. van Wijk, Alessia Vargiolu, Emmanuel Vega, Kimberley Velt, Jan Verheyden, Paul M. Vespa, Anne Vik, Rimantas Vilcinis, Victor Volovici, Nicole von Steinbüchel, Daphne Voormolen, Petar Vulekovic, Kevin K. W. Wang, Daniel Whitehouse, Eveline Wiegers, Guy Williams, Lindsay Wilson, Stefan Winzeck, Stefan Wolf, Zhihui Yang, Peter Ylén, Alexander Younsi, Frederick A. Zeiler, Veronika Zelinkova, Agate Ziverte, Tommaso Zoerle

**Affiliations:** 1 Section of Perioperative Medicine and Intensive Care, Department of Physiology and Pharmacology, Karolinska Institutet, Stockholm, Sweden; 2 School of Electrical Engineering and Computer Science, KTH Royal Institute of Technology, Stockholm, Sweden; 3 Neuroscience Intensive Care Unit, Department of Pathophysiology and Transplants, Fondazione IRCCS Cà Granda Ospedale Maggiore Policlinico, University of Milan, Milan, Italy; 4 Department of Biomedical Data Sciences, Leiden University Medical Center, Leiden, The Netherlands; 5 Division of Anaesthesia, Department of Medicine, University of Cambridge, Cambridge, UK; 6 Centre for Artificial Intelligence in Medicine, University of Cambridge, Cambridge, UK

**Keywords:** Traumatic brain injury, Endotypes, Intensive care unit, Critical care, Unsupervised clustering, Machine learning

## Abstract

**Background:**

While the Glasgow coma scale (GCS) is one of the strongest outcome predictors, the current classification of traumatic brain injury (TBI) as ‘mild’, ‘moderate’ or ‘severe’ based on this fails to capture enormous heterogeneity in pathophysiology and treatment response. We hypothesized that data-driven characterization of TBI could identify distinct endotypes and give mechanistic insights.

**Methods:**

We developed an unsupervised statistical clustering model based on a mixture of probabilistic graphs for presentation (< 24 h) demographic, clinical, physiological, laboratory and imaging data to identify subgroups of TBI patients admitted to the intensive care unit in the CENTER-TBI dataset (*N* = 1,728). A cluster similarity index was used for robust determination of optimal cluster number. Mutual information was used to quantify feature importance and for cluster interpretation.

**Results:**

Six stable endotypes were identified with distinct GCS and composite systemic metabolic stress profiles, distinguished by GCS, blood lactate, oxygen saturation, serum creatinine, glucose, base excess, pH, arterial partial pressure of carbon dioxide, and body temperature. Notably, a cluster with ‘moderate’ TBI (by traditional classification) and deranged metabolic profile, had a worse outcome than a cluster with ‘severe’ GCS and a normal metabolic profile. Addition of cluster labels significantly improved the prognostic precision of the IMPACT (International Mission for Prognosis and Analysis of Clinical trials in TBI) extended model, for prediction of both unfavourable outcome and mortality (both *p* < 0.001).

**Conclusions:**

Six stable and clinically distinct TBI endotypes were identified by probabilistic unsupervised clustering. In addition to presenting neurology, a profile of biochemical derangement was found to be an important distinguishing feature that was both biologically plausible and associated with outcome. Our work motivates refining current TBI classifications with factors describing metabolic stress. Such data-driven clusters suggest TBI endotypes that merit investigation to identify bespoke treatment strategies to improve care.

*Trial registration*

The core study was registered with ClinicalTrials.gov, number NCT02210221, registered on August 06, 2014, with Resource Identification Portal (RRID: SCR_015582).

**Supplementary Information:**

The online version contains supplementary material available at 10.1186/s13054-022-04079-w.

## Background

Traumatic brain injury (TBI) is a heterogeneous disease with a wide variety of injury mechanisms and tissue pathologies, affecting people at all stages of life. It is one of the leading causes of mortality and morbidity in young individuals globally, with a leading global cause being road traffic incidents (RTI) [1]. Additionally, the incidence of TBI in older patients is increasing as this multi-morbid and fall-prone population increases in prevalence [2].

Although mortality from TBI has decreased over the last 30 years, the proportion of patients with favourable outcomes have remained relatively unchanged [2–4], despite developments such as intracranial pressure (ICP) monitoring [5]. A recent report identified large variation in TBI management in a European multi-centre cohort, without a corresponding variation in outcomes [6]. While it is possible that these management variations truly had no impact on outcome, this result could also be due to a substantial heterogeneity of the disease masking treatment effect in relevant subgroups. Due to lack of high-quality evidence, variations in treatment strategies are based largely on local strategies rather than mechanistically aligned to injury types [7–9]. A better characterization of patients could allow discrimination into more specific and biologically relevant sub-groups based on clinical, biomarker, pathoanatomic, and physiological features.

This approach could provide a basis for determining whether specific treatments and interventions might be more effective in some of these sub-groups [7, 9–11]. However, implementation of such individualized treatment strategies relies on the identification of robust and relevant endotypes. Endotypes are subtypes of a clinical condition or syndrome, which can be characterized by distinct pathophysiology, and have an implicit likelihood of variation in response to therapies. This approach was first used to describe subgroups in asthma [12], but has now been used in other conditions [13]. Recently, unsupervised machine learning methods have been successful in discovering subgroups and endotypes with specific treatment-responses in diseases such as acute respiratory distress syndrome (ARDS) and sepsis in the intensive care unit (ICU) [14, 15].

The current classification of TBI simply as ‘mild’, ‘moderate’ and ‘severe’ is based on the level of consciousness at presentation, assessed using the Glasgow coma scale (GCS). While this is well known to be an important predictor of outcome and easy to operationalize, it is also clearly an overly simplistic description of such a complex disease and unlikely to be aligned with underlying pathobiology. As such, this simple classification provides a poor substrate from which to individualise care. Furthermore, it limits research into personalised medicine as populations stratified in this way retain biological heterogeneity and therefore are likely to be diverse in terms of their treatment response.

Instead, we hypothesize the existence of distinct clinically and/or physiologically determinable endotypes in patients with TBI requiring ICU treatment and that these may be described not only by canonical measures of injury severity, such as Glasgow Coma Scale (GCS), but also by pathophysiology. We further hypothesize that these parameters might present complex or nonlinear relationships to disease course and outcome, so that unsupervised/machine learning methods may be required to reveal underlying relationships between parameters. The aim of this study is not primarily to describe endotypes associated with outcome, but to describe endotypes that could motivate tailored treatments in the future, and potentially lead to improved outcome in patients with TBI.

## Methods

### Patient and feature selection

All patients over 18 years old enrolled in the multinational study Collaborative European Neuro Trauma Effectiveness Research in TBI (CENTER-TBI) ICU cohort (*N* = 2006) were included in the study, between 2014 and 2018 [4, 10], Additional file 1: Fig S1. All patients met the general inclusion criteria for CENTER-TBI (clinical diagnosis of TBI, presentation at hospital within 24 h from injury, and a clinical need for a CT scan) and were admitted to the ICU immediately after hospital admission. More than 2000 parameters were collected for each patient. Of these, a total of 35 early features were selected, including those in the core IMPACT prediction model for TBI [16] and features identified to be of clinical interest. The data and variables in the CENTER-TBI database were based on the synthesis of current knowledge of TBI in concert with much of European and Northern American expertise. The variables chosen for this analysis were early features with known or plausible relations with outcome, or deranged physiology (Table 1) as judged by clinicians with extensive neurointensive care experience. CT characteristics were extracted from a central imaging review. All selected features were recorded at presentation, i.e., either prehospital, during emergency room (ER) admission or early within the ICU, but no later than 24 h post-injury. Both GCS total and motor sub-score were included, as the total score is of clinical interest and the motor sub-score have shown prediction ability in the IMPACT model. Outcome was represented by the eight-point Glasgow outcome scale extended, (GOS-E) score, where GOS-E 1 = dead and GOS-E 8 = full recovery.

**Table 1:** Features included in the model

Age*	Hypotension	Sodium
Sex	MAP	Platelet count
ASA-PS classification	Heart rate	Creatinine
Anticoagulant or anti-platelet treatment pre-injury	Body temperature	Haemoglobin
BMI at arrival	SpO_2_	Rotterdam CT score
Type of injury	pH	Fisher classification
Cause of injury	Base excess	Midline shift (mm)
Pupillary reactivity*	PaO_2_	TAI
GCS motor score*	PaCO_2_	EDH
GCS total score	Lactate	aSDH
Hypoxia	Glucose	Contusion

All values were collected at admission

*ASA-PS* American society of anesthesiologists physical status classification; *BMI* body mass index; *GCS* Glasgow coma scale; *MAP* mean arterial pressure; *SpO*_*2*_ oxygen saturation; *PaO*_*2*_ arterial partial pressure of oxygen; *PaCO*_*2*_ arterial partial pressure of carbon dioxide; *TAI* traumatic axonal injury; *EDH* epidural hematoma; *aSDH* acute subdural hematoma

*Feature included in the IMPACT core model

Version 3.0 of the CENTER-TBI dataset was used for this work. Models were created using proprietary low-level code in C ++ and all other analyses were performed using R version 1.1.453 [17].

### The clustering model

We used a mixture of probabilistic graph models to construct an unsupervised classifier suitable for dealing with the mix of discrete and continuous features with missingness. The univariate probability distributions for all features were modelled as a product model, and compensating factors for each pair of strongly correlated features were included.

**Fig. 1:**
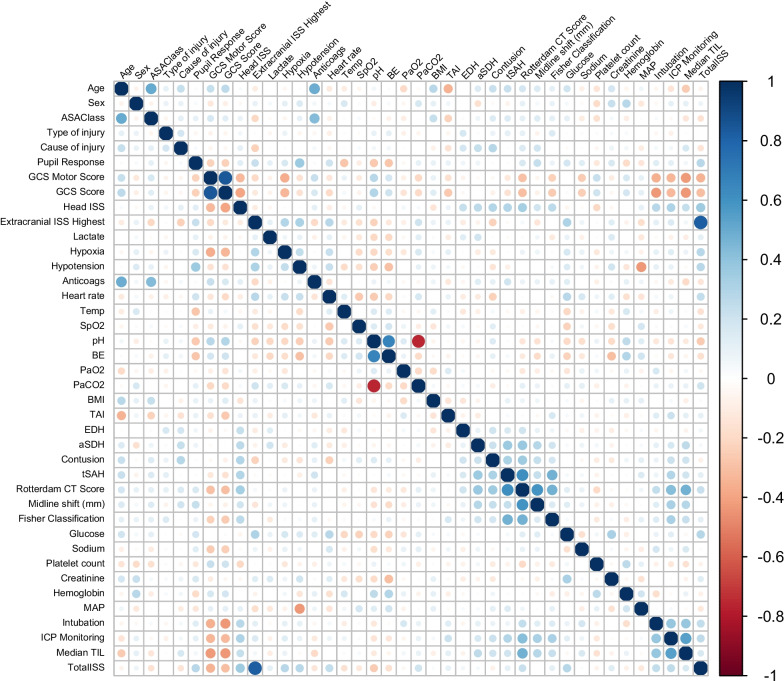
Linear correlation between all pairs of features. To visualize the strength of linear correlation between each pair of features, the value of the Pearson correlation coefficient is represented by the size and colour of the dots in the matrix. Strongly correlated features (pH and base excess, pH and arterial partial pressure of carbon dioxide (PaCO_2_), GCS motor and total score, Rotterdam CT score and midline shift, Rotterdam CT score and Fisher classification, GCS motor score and pupil response, age and ASA PS-class (American Society of Anesthesiologists physical status classification), and age and anticoagulants at baseline) were modelled as bivariate joint Gaussian distributions. GCS, Glasgow coma scale; ISS, injury severity score; SpO_2_, oxygen saturation; PaO_2_, arterial partial pressure of oxygen; PaCO_2_, arterial partial pressure of carbon dioxide; BMI, body mass index; TAI, traumatic axonal injury; EDH, epidural hematoma; aSDH, acute subdural hematoma; tSAH, traumatic subarachnoid haemorrhage; MAP, mean arterial pressure; ICP, intracranial pressure; TIL, therapy intensity level

To first determine which features were correlated and therefore would need to be considered jointly, linear correlations between features were examined graphically using the R package corrplot (version 0.84), Fig. 1 [18]. Pairs of strongly correlated features (pH and base excess, pH and arterial partial pressure of carbon dioxide (PaCO_2_), GCS motor and total score, Rotterdam CT score and midline shift, Rotterdam CT score and Fisher classification, GCS motor score and pupil response, age and ASA PS-class (American Society of Anesthesiologists physical status classification), and age and anticoagulants at baseline) were modelled as bivariate joint Gaussian distributions. Completeness of data is presented in, Additional file 2: Table S1.

To estimate the parameters and cluster membership probabilities in our graph mixture model, we used an expectation maximisation (EM) algorithm [19, 20]. This is a generalization of the maximum likelihood estimation of incomplete data and offers a principled, probabilistic approach to the unsupervised clustering of large multivariable datasets without the need for imputation when missingness is present [20]. Conceptually, the EM algorithm is a two-step iterative algorithm: in the expectation (*E*) step, the probability distribution over all clusters for each patient is calculated from the given parameters of the features in the cluster (i.e., the probability for cluster membership for each patient), and the maximization (*M*) step is the re-estimation of parameter distributions in all clusters. These steps are repeated until convergence, giving the most probable separation of clusters given the chosen number of clusters and predictor features. Further mathematical details are described in Additional file 3.

### Determination of number of clusters

We used a cluster stability to robustly determine the most appropriate number of clusters to choose [21]. Numbers from three to fifteen clusters were evaluated for stability. This selection was a clinical trade off – too many clusters might not be clinically relevant, despite the risk that they may represent potentially important separation of phenotypes. However, within this range a methodologically principled optimum may be identified.

**Fig. 2:**
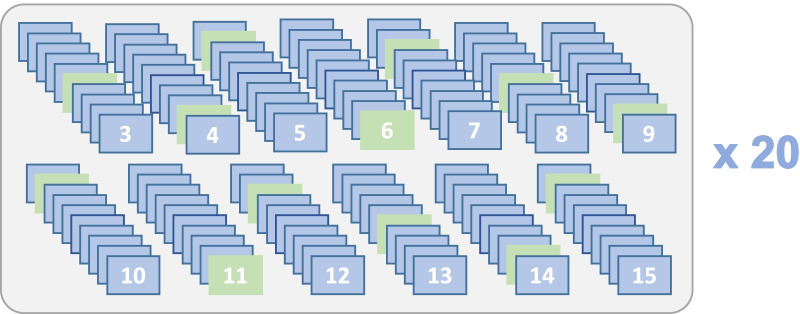
Ten models of each number of clusters between three to fifteen were created. The model with the highest log likelihood was chosen as the best model. This was repeated twenty times. Median, minimum, and maximum cluster similarity index (CSI, defined as the fraction of patients assigned to the same cluster in two models), of the twenty models were calculated. The median CSI is presented in Fig. 3

For each number of clusters considered, we created ten different models, using different random seeds. The log-likelihood for each model was calculated, and the model with the highest log-likelihood was selected. This process was repeated twenty times (Fig. 2) and cluster similarity was quantified using a cluster similarity index (CSI) defined as the fraction of patients who were assigned to the same cluster in two models [21]. CSI was calculated between all pairs of the models of the same number of clusters, and median and interquartile range (IQR) was calculated. As the CSI, when numbers of clusters < < number of patients, by nature is higher for lower number of clusters, a penalty for the number of clusters was added by subtracting 1/*n* clusters from all median CSI. The optimal clustering was defined as number of clusters with the highest median CSI (representing the most stable number of clusters). When describing the clusters, the model of the optimal number of clusters with the highest log-likelihood was chosen to represent our model.

### Evaluation of the clusters

To investigate the importance of each feature for the model, the mutual information (MI) was calculated between each feature and the cluster labels. The MI represents how well the cluster label is determined by a feature, with respect to how the distributions differ between the clusters. Features were considered to be of value if the MI > 0.1. A descriptive analysis of the clusters using these features was undertaken. Univariable logistic regression analysis was performed to determine the pseudo-explained variance between cluster label and outcome, and a multivariable regression analysis was performed to investigate if the cluster label could improve predicted outcome over the “International Mission for Prognosis and Analysis of Clinical Trials in TBI” (IMPACT) variables which have been well characterised as predictors in TBI [16]. For the outcome prediction (but not the clustering), missing values were imputed using the multiple imputation with chained equations (MICE) algorithm in *R* [22]. The observed mortality and unfavourable outcome (defined as GOS-E < 5) frequencies in all clusters was compared to the IMPACT predicted outcome.

## Results

### Patient characteristics

278 patients were excluded due to missing Glasgow outcome scale extended (GOS-E) score at 6 months, leaving 1728 patients for the analysis. The mean age was 50.4 years (SD 19.3) and 1269 (73.4%) were male. The most common causes of injury were RTIs (46.5%) and falls (43.7%). The overall mortality in the cohort was 22%, and 45% had unfavourable outcomes (defined as Upper Severe Disability or worse according to the GOS-E outcome scale) 6 months post-injury. Based on the IMPACT core model, the overall analysis cohort had a predicted mortality of 31%, and a predicted unfavourable outcome of 51%.

### Optimal number of clusters

**Fig. 3:**
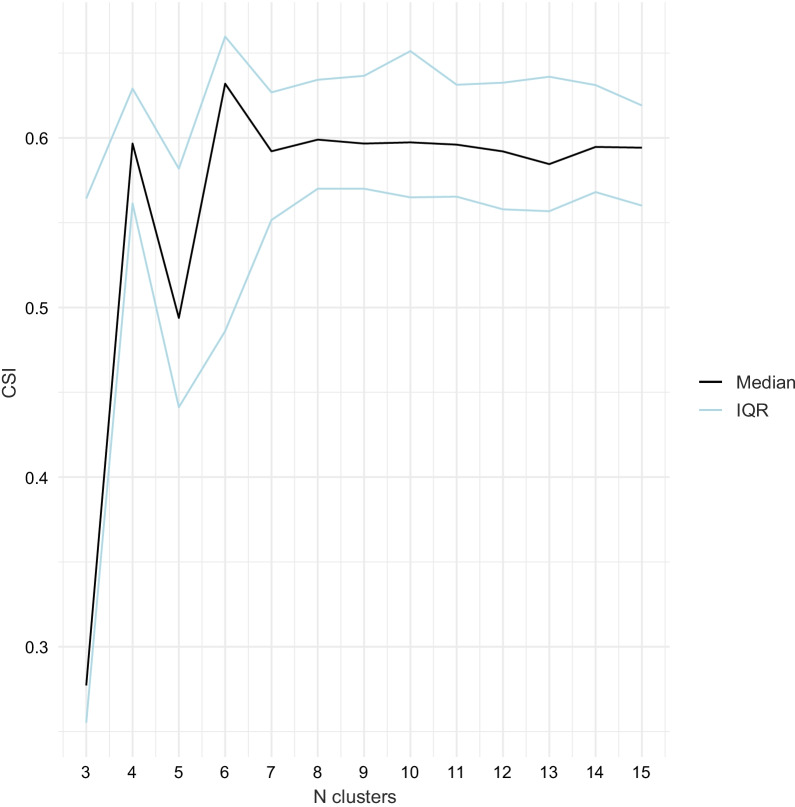
Median, minimum, and maximum cluster similarity index (CSI) of 20 models for each number of clusters. A penalty for the number of clusters was added by subtracting 1/n clusters from the CSI values. Median CSI = 1 indicates perfect match, 0 indicates no matches between different models

**Fig. 4:**
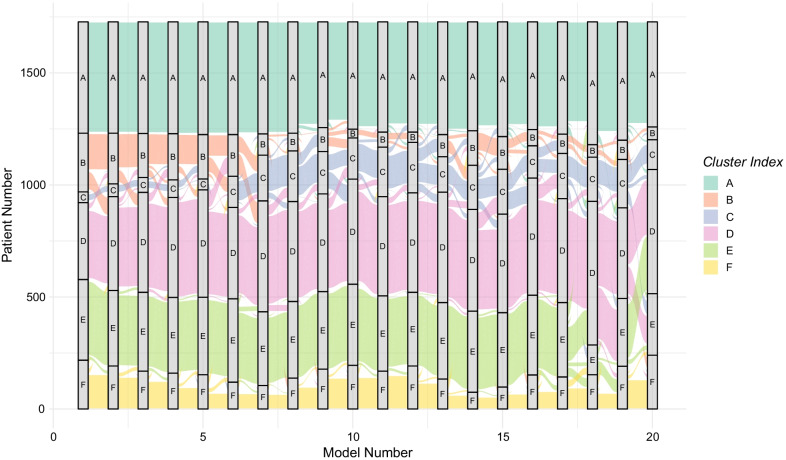
Visualization of model stability. The cluster each patient belongs to in twenty randomly created different models is visualized for each of the twenty models. The models are aligned with respect to highest log likelihood, from left to right

Applying a penalty of 1/*n* from the median CSI of each number of clusters revealed a peak in median CSI, indicating the highest cluster stability, for 6 clusters, Fig. 3. Cluster assignments in twenty randomly generated models of 6 clusters are presented in Fig. 4, demonstrating the robust reproducibility of our model. The number of patients in the clusters was 48, 262, 360, 343, 218, and 497, respectively.

### Importance of features included in the model

**Fig. 5:**
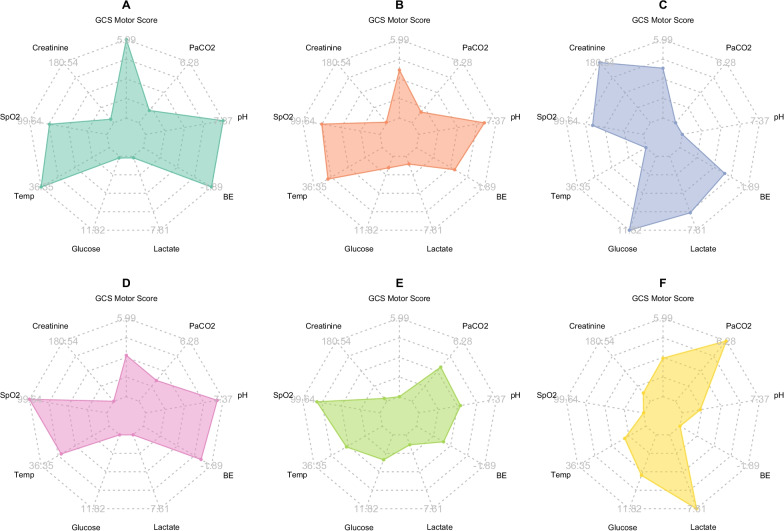
Features with highest mutual information (MI) for all clusters. The axes range from minimum to maximum of cluster averages for each feature. GCS, Glasgow coma scale; PaCO_2_, arterial partial pressure of carbon dioxide; SpO_2_, oxygen saturation

**Fig. 6:**
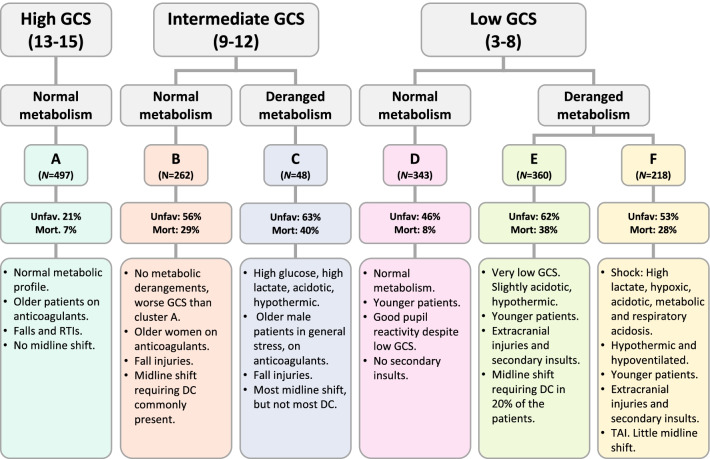
Description of the 6 clusters. The six identified clusters can, in general, be seen as distinguished by GCS and degree of metabolic derangement. The percentage of patients in each cluster with unfavourable outcome and cluster mortality is indicated as well. RTIs, road traffic incidents; DC, decompressive craniectomy; TAI, traumatic axonal injury

GCS motor score, GCS total score, lactate, oxygen saturation (SpO_2_), creatinine, glucose, base excess, pH, PaCO_2_ and body temperature were identified as the most important features in our model with respect to MI. Median values in all clusters are presented in Fig. 5, Table 2, and a full list of MI and cluster medians for all features is provided in, Additional file 4: Table S2. A description of cluster characteristics is given in Fig. 6 and Table 3. The results were interpreted by the authors with extensive academic and clinical neurointensive care experience. The six clusters may generally be described by combinations of GCS score and degrees or patterns of deranged metabolism. Outcome predictions and parameters, as well as injury severity and treatment features which were not used in the clustering, are presented in Table 4.

**Table 2:** Cluster medians and mutual information (MI) for features with MI > 0.1

Cluster	All patients	A	B	C	D	E	F	MI
*N* patients	1728	497	262	48	343	360	218	
GCS motor Score	5 (1–6)	6 (6–6)	5 (2.5–5)	5 (3.5–5)	4 (2–5)	1 (1–1)	4 (1–5)	1.44
GCS total Score	9 (4–14)	15 (14–15)	9 (6–12)	9 (6.75–13)	7.5 (6–10)	3 (3–3)	7 (4–10)	1.29
Lactate [mmol/L]	2.2 (1.4–3.4)	2.0 (1.2–2.7)	2.3 (1.4–3.4)	4.9 (2.3–8.1)	1.7 (1.2–2.4)	2.2 (1.4–3.4)	5.3 (2.9–10)	0.88
SpO_2_ [%]	99 (96–100)	98 (96–100)	98 (96–100)	98 (95–100)	100 (99–100)	99 (97–100)	95 (85–98)	0.69
Creatinine [µmol/L]	75 (62–89)	76 (64–88)	70 (58–86)	106 (64–257)	71 (60–83)	74 (59–91)	83 (71–101)	0.63
Glucose [mmol/L]	7.7 (6.5–9.4)	7.2 (6.3–8.4)	8.0 (6.7–9.3)	8.5 (6.9–14.3)	7.3 (6.3–8.6)	8.1 (6.8–10.5)	9.1 (6.9–11.8)	0.25
Base Excess [mmol/L]	− 2.9 (− 5.7–0.9)	− 1.7 (− 3.7 to − 0.2)	− 3.15 (− 5.3 to − 1.1)	− 3.9 (− 12.1–0.6)	− 2.3 (− 4 to − 1)	− 3.6 (− 6.6 to − 1)	− 5 (− 7.9 to − 2)	0.23
pH	7.35 (7.28–7.39)	7.37 (7.32–7.41)	7.35 (7.31–7.4)	7.27 (7.09–7.4)	7.36 (7.32–7.39)	7.32 (7.25–7.39)	7.29 (7.20–7.36)	0.23
PaCO_2_ [kPa]	5.5 (4.8–6.2)	5.3 (4.8–6)	5.3 (4.7–6)	5.3 (4.4–5.8)	5.4 (5–6)	5.6 (4.8–6.7)	5.9 (5–7.2)	0.14
Body temperature [°C]	36.0 (35.4–36.7)	36.5 (35.9–36.9)	36.2 (35.5–36.7)	35.7 (34.3–36.6)	36 (35.4–36.6)	35.9 (35.0–36.6)	35.8 (34.8–36.4)	0.12

Data presented as median (interquartile range)

*GCS*, glasgow coma scale; *SpO*_*2*_, oxygen saturation; *PaCO*_*2*_, arterial partial pressure of carbon dioxide; *MI*, mutual information

**Table 3:** Narrative description of typical physiological and clinical features of the clusters identified

Cluster	Cluster description	Other typical characteristics	Mortality %
A	Mild TBI. No metabolic derangements	Older patients with anticoagulation. Falls/RTIs. No midline shift	7
B	Moderate TBI. No metabolic derangements	Older females with anticoagulation. Fall injuries. Midline shift requiring DC commonly present	29
C	Moderate TBI. High glucose, high lactate, acidotic, hypothermic	Older males with anticoagulation. Fall injuries. Most midline shift but mostly not requiring DC	40
D	Severe TBI. No metabolic derangements	Younger patients. RTIs. Good pupil reactivity, severe TBI. No secondary insults	18
E	Severe TBI. Very low GCS at arrival. Slightly acidotic, hypothermic	Younger patients. RTIs. High Rotterdam CT score, midline shift leading to DC in 20% of patients. Extracranial injuries. Secondary insults	38
F	Severe TBI. High lactate, hypoxic, acidotic, hypoventilation. Metabolic and respiratory acidosis. Hypothermic	Younger patients in shock with extracranial injuries and secondary insults. TAI. No midline shift	28

*TBI* traumatic brain injury; *DC* decompressive craniectomy; *GCS* glasgow coma scale; *RTI* road traffic incident; *TAI* traumatic axonal injury

**Table 4:** Injury severity, outcome and predicted outcome with the IMPACT lab model

	All patients	A	B	C	D	E	F
Age	52 (33–67)	53 (36–67)	56.5 (42–69)	58 (48–74.5)	52 (29–67)	47.5 (32–62)	44 (29–62)
Male sex	1269 (73.4)	370 (74.4)	177 (67.6)	40 (83.3)	241 (70.3)	277 (76.9)	164 (75.2)
Decompressive Craniectomy	216 (12.5)	22 (4.4)	48 (18.3)	7 (14.6)	40 (11.7)	73 (20.3)	26 (11.9)
ISS	29 (25–41)	25 (16–34)	26 (25–38)	29.5 (25–41.5)	29 (25–41)	38 (25–52.5)	38 (25–50)
Head ISS	25 (16–25)	16 (9–16)	25 (16–25)	25 (16–25)	25 (16–25)	25 (25–25)	25 (16–25)
Highest extracranial ISS	9 (0–16)	4 (0–16)	4 (0–9)	9 (0–10.75)	9 (0–16)	9 (0–16)	9 (0.25–16)
Intubation	1334 (78.9)	218 (45.7)	224 (85.8)	39 (84.8)	314 (93.2)	347 (97.5)	192 (89.7)
ICP monitoring	757 (44.2)	76 (15.6)	153 (58.4)	19 (40.4)	177 (51.8)	225 (62.7)	107 (49.5)
Median daily TIL	2 (0–5.5)	0.5 (0–1.5)	3.5 (0.5–8)	2 (0–3.25)	3 (1–5)	4.5 (2–8.5)	3 (1–5.625)
*GOS-E at 6 months*							
1	388 (22.5)	34 (6.8)	77 (29.4)	19 (39.6)	62 (18.1)	135 (37.5)	61 (28)
2 or 3	268 (15.5)	40 (8)	50 (19.1)	5 (10.4)	70 (20.4)	68 (18.9)	35 (16.1)
4	123 (7.1)	31 (6.2)	19 (7.3)	6 (12.5)	27 (7.9)	21 (5.8)	19 (8.7)
5	241 (13.9)	71 (14.3)	34 (13)	5 (10.4)	49 (14.3)	47 (13.1)	35 (16.1)
6	214 (12.4)	70 (14.1)	31 (11.8)	5 (10.4)	51 (14.9)	29 (8.1)	28 (12.8)
7	229 (13.3)	107 (21.5)	24 (9.2)	2 (4.2)	40 (11.7)	37 (10.3)	19 (8.7)
8	265 (15.3)	144 (29)	27 (10.3)	6 (12.5)	44 (12.8)	23 (6.4)	21 (9.6)
Mortality, %	22	7	29	40	18	38	28
Unfavourable outcome, %	45	21	56	63	46	62	53
IMPACT predicted mortality, mean (SD)	27	13 (8)	27 (18)	30 (23)	24 (16)	45 (18)	32 (22)
IMPACT predicted unfavourable outcome, mean (SD)	45	26 (15)	46 (24)	48 (27)	43 (22)	70 (18)	51 (26)
Difference between predicted and observed mortality, %	5	6	− 2	− 10	6	7	4
Difference between predicted and observed unfavourable outcome, %	0	5	− 10	− 15	− 3	8	− 2

Data presented as median (IQR) or *N* (%) if not else is stated

*ISS* injury severity score; *ICP* intracranial pressure; *TIL* therapy intensity level; *GOS-E* glasgow outcome scale extended; *IMPACT* international mission on prognosis and clinical trial design in TBI

### Relation of clusters to outcome

Outcome information was not included in the clustering process. In all clusters except Clusters B and C, the IMPACT model overestimated mortality with over-estimation ranging from 4 to 7%, but underestimated functional outcome in four of the six clusters, with an underestimation ranging from − 2 to − 15%. By adding the cluster label to the IMPACT extended model variables (age, GCS motor score, pupil reactivity, Rotterdam CT score, presence of traumatic subarachnoid haemorrhage, intraventricular haemorrhage, epidural hematoma, hypoxia, and hypotension), predictions for mortality as well as unfavourable outcome were improved with a small but statistically significant increase of Nagelkerke pseudo-*R*^*2*^ from 0.42 to 0.44 and from 0.36 to 0.38, respectively (*p* = 0.001 and *p* = 2.9 × 10^–5^, respectively). The improvement in explained variance was comparable to that achieved by the addition of laboratory values within the original IMPACT model for mortality prediction (Nagelkerke pseudo-*R*^2^ 0.42 to 0.44, *p* = 3.6 × 10^–5^), and prediction of unfavourable outcome (Nagelkerke pseudo-*R*^2^ of 0.36 to 0.37, *p* = 2.1 × 10^–4^). These clusters, therefore, appear to represent groups with outcomes that differ in both directions from current prediction models. The relationships of clusters to outcomes and IMPACT predicted outcomes are seen in Table 4.

## Discussion

We have used an EM clustering approach, based on early clinical and laboratory data, that identified six distinct potential clusters of TBI patients admitted to the ICU. These clusters exhibited distinct systemic metabolic profiles defined by combinations of plasma lactate, SpO_2_, creatinine, glucose, base excess, pH, PaCO_2_, and body temperature, which in combination with GCS, characterizes 6 clinically distinct patient endotypes.

Profiles of metabolic derangement may be readily recognized clinically, and arguably contribute to our impression of severity state in TBI patients in the ICU. However, except for blood glucose, the identified features are not incorporated into current formal definitions of TBI severity or outcome prediction models, although earlier publications have reported improved accuracy adding physiology-based prediction scores, such as APACHE II score (Acute Physiology and Chronic Health Evaluation II) [23, 24].

We hypothesize there to be several diverging mechanisms leading to deranged metabolism in TBI patients that are not fully captured by conventional ICU disease severity metrics, such as APACHE II scores. These may include interplay of secondary and extracranial injury, and concurrent comorbidities. This is the rationale for defining metabolic profiles using several features highly correlated to pH– base excess, PaCO_2_, and lactate that may reflect several intrinsic mechanisms. In Cluster *C*, a deranged metabolic picture appears to reflect a general stress response, with high lactate and high blood glucose in more elderly patients prone to insulin resistance. In contrast, Cluster *E* is representative of younger patients displaying tachycardia and relative hypotension, in whom the cause of metabolic compromise is more likely to reflect a state of systemic shock, which is more likely to be related to extracranial injury. It must be noted that the endotype with a general stress response is a relatively small subset of patients (*N* = 48) but may nevertheless be of clinical importance as it seems likely to result from a distinct pathology. These two metabolic pictures may easily be distinguished clinically and likely benefit from different treatment approaches allowing for articulation of broad strategies of care and overall management in endotypic groups.

Although GCS has been shown to be one of the most principal factors in classification of TBI [16], the weakness of GCS alone as a classifier of TBI severity becomes apparent in this study. In Cluster *A*, comprising 28% (*N* = 497) of the total number of patients TBI severity would be classified as ‘mild’ based on GCS. This group was in general characterized by patients who were older with comorbidities and receiving anticoagulant or antiplatelet treatments pre-injury and the cause of ICU admission did not seem to be explained by extracranial injuries, Table 4, but may rather have been motivated by a need for clinical observation, something which was highlighted in a previous CENTER-TBI sub-study [6]. However, the morbidity and treatment burden in this group is substantial: 45% of patients in this cluster were intubated, 15% had ICP monitoring, and 7% died within 6 months post-injury. In addition, Cluster *C*, the cluster with deranged metabolism, had the largest deviation in outcome prediction in comparison with the IMPACT model. When compared to Cluster *D* (which comprises patients with severe TBI but without such metabolic derangement), Cluster *C* had a worse outcome, which further supports the impact of assessing the metabolic profile in TBI patients, beyond derangements that are simply explained by extracranial injuries. It may also reflect an increased vulnerability of the brain in older patients which is not captured in other factors associated with severity of brain damage, such as GCS. Although they did not have as complete a description of biochemical derangements in their dataset, Folweiler et al. elegantly showed TBI clustering that did not relate well to ‘mild’, ‘moderate’ or ‘severe’ descriptions of TBI [25]. In our study again, although GCS is here shown to be an important component of endotypes in an ICU cohort, metabolic profiles may add additional, clinically important, information as descriptors of TBI severity, and perhaps identify patient groups in which treatment should be individualized.

Surprisingly, neither our model nor earlier endotypic multidimensional descriptions of TBI patients generated by unsupervised machine learning methods have identified the type of intracranial injuries and CT characteristics as relevant for describing endotypes [25, 26]. However, a recent study could identify clusters based solely on CT characteristics [27], supporting that these factors may play an essential role in understanding the type of injury and determining the need for intracranial surgery, and prediction models using CT findings such as the Marshall, Rotterdam, Helsinki and Stockholm CT scores do discriminate outcome [28–31]. These findings are less evident in multivariable analyses when including GCS and other IMPACT variables as covariates, then contributing approximately only 5% additional pseudo-variance toward outcome [31, 32]. This covariance may be a possible explanation as to why we could not identify CT characteristics as one of the most important discriminative factors between the clusters.

Unsupervised learning is appealing from the point of view of objectivity, but cannot be performed entirely without making certain choices, and requires subsequent interpretation. The number of clusters is a trade-off between not being overwhelmed by multitudes of clusters with small sizes that cannot be interpreted, and very few clusters inherently containing little discriminant information. The identification of six clusters of TBI patients was supported by both the maximal and stable reproducibility represented by a CSI of 80%, as well as a suggestion of clinical relevance. Most clusters were relatively stable across different random initializations of the clustering, with the exception of cluster *B* and *C*, both representing patients with intermediate GCS, Fig. 4. By nature, more extreme patient characteristics and their corresponding clusters tend to be more stable while the intermediate level characteristics and clusters are less stable. Most patients were clearly assigned to a stable cluster, as seen in, Additional file 1: Fig. S2. It is unrealistic to expect perfectly stable cluster assignment in heterogenous real world data with any method, particularly with random assignment to initial clusters. We believe our evaluation of model robustness to be an important and generalisable strength of our work.

In this study we are naturally limited by the variables collected. These represent nevertheless the compound experience and knowledge of a large cohort of leading TBI researchers and clinicians during the planning of the study. However, additional variables such as future biomarkers and genetic profiles may be needed to sufficiently describe patient heterogeneity in TBI. Furthermore, despite that the object of clustering is to identify reproducible compound and complex patterns, it does not weigh variables toward severity as would for example an experienced clinician and represents a general limitation of unsupervised leaning.

The aim of this study was not primarily to create clusters of TBI patients toward outcome prediction, but to identify clinically relevant and distinct endotypes of patients, which could potentially infer personalization of future treatment strategies. Current TBI therapy is based on limited high-level evidence, leading to between-centre treatment variability beyond that of case mix [7, 9, 33]. Further discrimination of patient heterogeneity has been identified as necessary to further the field [7, 9]. Prediction of both mortality and functional outcome using the IMPACT extended model was significantly improved by adding cluster labels. That the metabolic cluster profiles identified in this study are significantly associated with outcome, despite an unsupervised clustering method (not including outcome), supports a biological underpinning and motivates further investigation. A natural progression will be to investigate if the clusters described in this study exhibit a different temporal trajectories in the ICU or, in analogy with work within the field of ARDS [14] respond differently to treatments in earlier RCTs.

## Conclusions

While GCS is a strong predictor of TBI outcome, an admission metabolic profile incorporating hypothermia, lactatemia, blood glucose, SpO_2_, PaCO_2_, pH, base excess and creatinine allows for a more holistic description of patients with TBI who require ICU care. Synthesis of these data using an unsupervised clustering method reveals six distinct and stable subgroups of TBI patients. Although not a key objective of the analysis, we found that clusters contain information that can provide a significantly better explanation of outcome beyond that provided by variables used in current outcome prediction models. The addition of biomarkers and genetics may improve this endotypic classification further. Future studies should address replication and validation of this approach, but our work provides an important starting point from which to devise and prospectively investigate therapeutic strategies individualised to more biologically relevant groups or TBI patients.

## Supplementary Information

**Additional file 1**: Figures of flowchart of patient selection and cluster assignment probabilities.

**Additional file 2**: Table of completeness of all features in the dataset.

**Additional file 3**: Additional details of the clustering method.

**Additional file 4**: Table of Cluster means and overall means, min, max and standard deviation of all features.

## Data Availability

CENTER-TBI encourages data sharing, and there is a data sharing statement published. Data will be made available to researchers who provide a study proposal that is approved by the management committee to achieve the aims in the approved proposal. Proposals can be submitted online at https://www.center-tbi.eu/data/sharing. A data access agreement is required, and all access must comply with regulatory restrictions imposed on the original study.

## Abbreviations

APACHE II score: Acute physiology and chronic health evaluation II score
ARDS: Acute respiratory distress syndrome
ASA-PS class: American society of anesthesiologists physical status classification
aSDH: Acute subdural hematoma
BMI: Body mass index
CENTER-TBI: Collaborative European neuro trauma effectiveness research in TBI
CSI: Cluster similarity index
DC: Decompressive craniectomy
EDH: Epidural hematoma
EM: Expectation maximization
GCS: Glasgow coma scale
GOS-E: Glasgow outcome scale extended
ICU: Intensive care unit
ICP: Intracranial pressure
IMPACT: International mission for prognosis and analysis of clinical trials in TBI
IQR: Interquartile range
ISS: Injury severity score
MAP: Mean arterial pressure
MI: Mutual Information
MICE: Multiple imputation with chained equations
PaCO_2_: Arterial partial pressure of carbon dioxide
PaO_2_: Arterial partial pressure of oxygen
RTC: Road traffic collision
RTI: Road traffic incident
SpO_2_: Oxygen saturation
TAI: Traumatic axonal injury
TBI: Traumatic brain injury
TIL: Therapy intensity level
tSAH: Traumatic subarachnoid haemorrhage
