# Supplementary material for: Clustering identifies endotypes of traumatic brain injury in an intensive care cohort: a CENTER-TBI study
Source: Crit Care. 2022 Jul 27;26:228. doi: 10.1186/s13054-022-04079-w (PMC9327174; doi:10.1186/s13054-022-04079-w)
Supplement: Supplementary file 1 — Additional file 1: Figures of flowchart of patient selection and cluster assignment probabilities. [file 13054_2022_4079_MOESM1_ESM.docx]

**Patients included in analysis**

***N=1,728***

Missing outcome at 6 months

*N=278*

<18 years old

*N=132*

*N=2,006*

Patients in ICU stratum

*N=2,138*

Patients enrolled in CENTER-TBI core study

*N=4,509*

Patients in ER or Admission stratum

*N=2,371*

**Supplementary Fig 1: Flowchart of patient selection for the analysis.** ER, Emergency room; ICU, Intensive care unit.


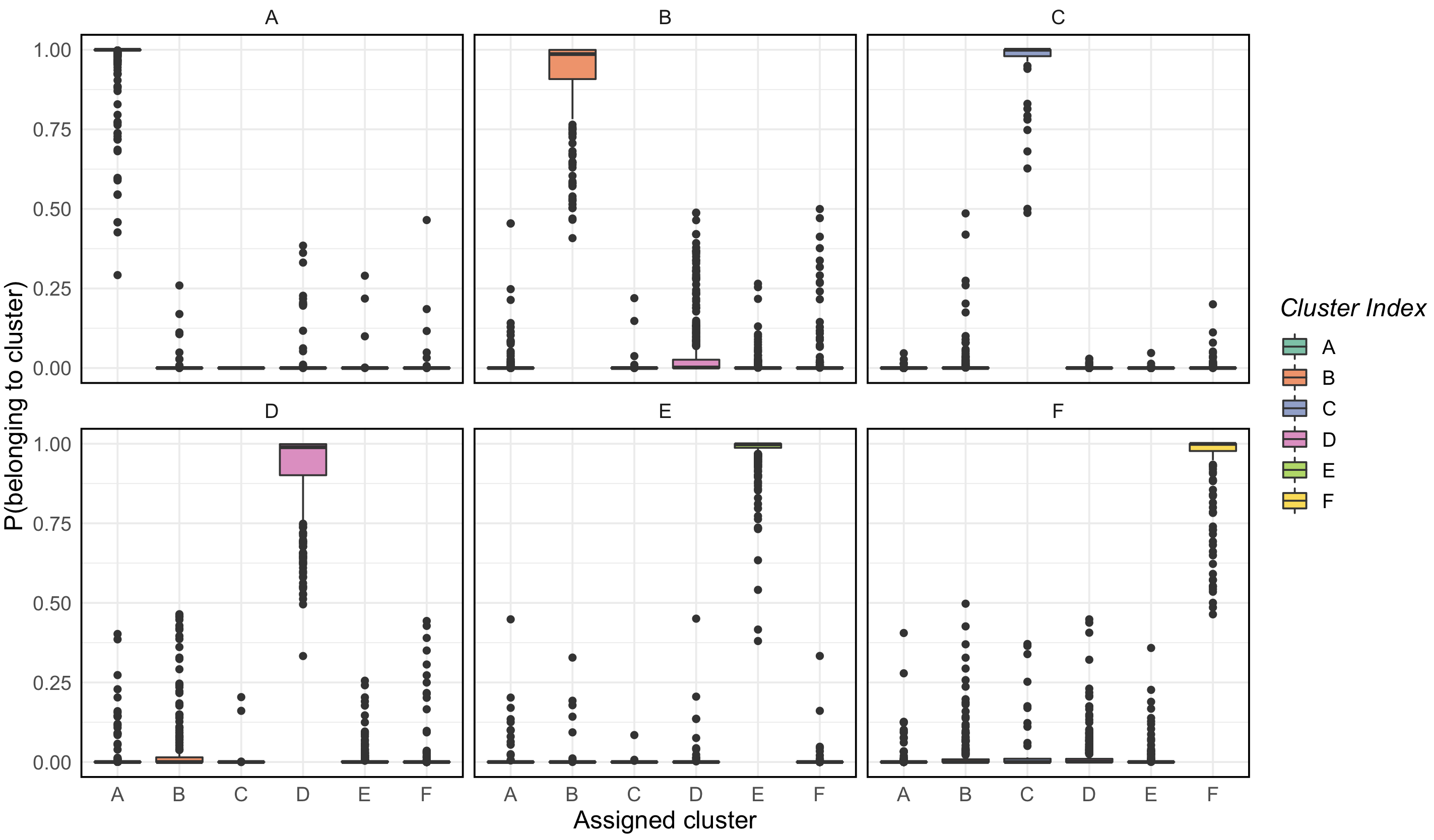


**Supplementary Fig 2: Cluster assignment stability.** Each subplot represents the probability of belonging to that cluster, stratified by the assigned cluster. Most patients had a very high probability of belonging to the assigned cluster, while the probability of belonging to another cluster was low.
