# Supplementary material for: Clustering identifies endotypes of traumatic brain injury in an intensive care cohort: a CENTER-TBI study
Source: Crit Care. 2022 Jul 27;26:228. doi: 10.1186/s13054-022-04079-w (PMC9327174; doi:10.1186/s13054-022-04079-w)
Supplement: Supplementary file 2 — Additional file 2: Table of completeness of all features in the dataset. [file 13054_2022_4079_MOESM2_ESM.docx]

| Supplementary Table 1. Completeness of features included in the analysis. | |
| --- | --- |
| **Feature** | ***Completeness, %*** |
| Lactate | 46 |
| Body temperature | 47 |
| Base Excess | 50 |
| pH | 54 |
| PaO_2_ | 54 |
| PaCO_2_ | 54 |
| BMI at arrival | 69 |
| Glucose | 78 |
| Platelet count | 82 |
| Creatinine | 82 |
| Sodium | 85 |
| Haemoglobin | 85 |
| SpO_2_ | 87 |
| TAI | 87 |
| EDH | 87 |
| aSDH | 87 |
| Contusion | 87 |
| Rotterdam CT Score | 88 |
| Fisher Classification | 88 |
| Heart rate | 89 |
| Mean arterial pressure | 90 |
| Cause of injury | 92 |
| Anticoagulant or anti-platelet treatment pre-injury | 93 |
| Pupillary reactivity | 94 |
| GCS Total Score | 94 |
| ASA-PS classification | 95 |
| GCS Motor Score | 97 |
| Type of injury | 98 |
| Hypoxia | 99 |
| Hypotension | 99 |
| Midline Shift (mm) | 100 |
| Age | 100 |
| Sex | 100 |
| PaO_2_, Arterial partial pressure of oxygen; PaCO_2_, Arterial partial pressure of carbon dioxide; BMI, Body Mass Index; SpO_2_, Oxygen saturation; TAI, Traumatic axonal injury; EDH, Epidural hematoma; aSDH, Acute subdural hematoma; GCS, Glasgow Coma Scale; ASA-PS classification, American Society of Anesthesiologists physical status classification. | |
