# Supplementary material for: Clustering identifies endotypes of traumatic brain injury in an intensive care cohort: a CENTER-TBI study
Source: Crit Care. 2022 Jul 27;26:228. doi: 10.1186/s13054-022-04079-w (PMC9327174; doi:10.1186/s13054-022-04079-w)
Supplement: Supplementary file 3 — Additional file 3: Additional details of the clustering method. [file 13054_2022_4079_MOESM3_ESM.docx]

# THE CLUSTERING MODEL

We used a mixture of probabilistic graph models to construct an unsupervised classifier suitable for dealing with the mix of discrete and continuous variables with missingness. In a probabilistic graph model, the joint probability distribution over all input features is written as a product expansion, which means that it is expressed as a product involving probability distributions of just one or a few variables each. The simplest case occurs if it can be assumed that all features are independent within each cluster. Then a pure product model can be used, which consist of a product of all the univariate feature distributions. However, as some of the features in this domain are strongly correlated, compensating factors for each such pair of correlated features need to be included in the product, in the form of P(A,B)/(P(A)P(B)).

The resulting mixture model has several advantages over more commonly used mixture models (such as a Gaussian mixture model): It can handle a mix of discrete and continuous variables since the separate distributions are just multiplied together, and the number of training data required is small due to the low order of the involved distributions. For the same reason, no imputation of missing values is needed since a missing value only affects its own distribution and all other values in the sample can be fully exploited which is an advantage over previous attempts at unsupervised learning.

In this paper we adopt the Bayesian convention of using *P*() both for discrete probabilities and for probability density functions. Indeed, in most equations below, *x_i_* may represent either a discrete variable or a continuous valued variable, and the expression *P*(*x_i_*) should be interpreted accordingly.

As our cluster model we use a mixture of probabilistic graph models. This means that the complete distribution over the observed features ***X*** is written as a weighted sum:

$P\left( \boldsymbol{X} \right)=\sum_{j} \pi_{j}P\left( \boldsymbol{X} \right|c_{j})$ (1)

Where *c_j_* indicates that the cluster index is *j* and *πj* is the proportion of the cluster in the whole distribution (i.e., the probability of any sample to belong to that cluster). Each cluster component in the sum is in turn modelled as a probabilistic graph. This means that the joint distribution over ***X*** is expressed as a product of powers of distributions over subsets of the features. In the simplest case, if we can assume that all features are statistically independent of each other within each cluster, then we can write the joint distribution for that cluster as a product of the individual distributions over each feature *x_i_*:

$P\left( \boldsymbol{X} \right|c_{j})= \prod_{i} P\left( x_{i} \right| c_{j})$ (2)

If the features are not statistically independent, we need to add compensating factors to this product. In this work we follow the approach of Chow and Liu,^1^ of building a maximum spanning dependency tree over the features, adding edges between the most correlated features (above a threshold) while avoiding to create cycles in the graph. Then we can express the joint distribution for the cluster as:

$P\left( \boldsymbol{X} \right|c_{j})= \prod_{i} P\left( x_{i} \right| c_{j}) \prod_{(i^{'},i^{''})\in L} \frac{P\left( x_{i'} , x_{i''} \right| c_{j})}{P\left( x_{i'} \right| c_{j})P\left( x_{i''} \right| c_{j})}$ (3)

where *L* is the set of pairs of features (*i’*, *i’’*) forming edges in the maximum spanning dependency tree. Technically we could use a separate dependency tree for each cluster. However, here we have used the same dependency tree in all clusters, which was constructed by using the pairwise correlations in the full data set.

One reason to use this model is that it can handle missing values, avoiding the need for imputation. Whenever a feature value is missing, the factors related to that feature are just omitted in the product above.

The second reason for using this model is that it can handle a mix of discrete and continuous valued features. If *x_i_* is a discrete variable, the parameters are the probabilities for the possible outcomes of *x_i_*:

$P\left( x_{i}=v \right| c_{j})=p_{jiv}$ discrete *x_i_* (4)

If *x_i_* is a continuous valued variable, it is modelled as a Gaussian distribution with mean *μ_ji_* and standard deviation *σ_ji_* as parameters:

$P\left( x_{i} \right| c_{j})= \frac{e^{-{{(x_{i}-\mu_{ji})}^{2}}/{(2\sigma_{ji}^{2})}}}{\sqrt{2\pi\sigma_{ji}}}$ continuous *x_i_*  (5)

The expressions for the joint distributions of a pair of features are analogous in the case of two discrete features or two continuous features:

$P\left( x_{i^{'}}=v, x_{i^{''}}=w \right| c_{j})=p_{ji^{'}i^{''}vw}$ discrete x_i’_ and x_i’’_ (6)

$P\left( x_{i^{'}}, x_{i^{''}} \right| c_{j})= \frac{e^{-{(\boldsymbol{x}_{\boldsymbol{l}}-\boldsymbol{\mu}_{jl})}^{'}\sum_{jl}^{-1} (\boldsymbol{x}_{l}-\boldsymbol{\mu}_{jl})/2}}{\sqrt{2\pi|\Sigma_{jl}|}}$ continuous x_i’_ and x_i’’_ (7)

where *l* represents the pair (i^’^, i^’’^) and ***x****_l_* is a vector with *x_i’_* and *x_i’’_* as elements.

If one feature in a pair is discrete and the other continuous, the joint distribution is expressed as:

$P\left( x_{i^{'}}=v, x_{i^{''}} \right|c_{j})=p_{ji^{'}v}\frac{e^{{-{(x_{i^{''}}-\mu_{ji^{'}vi^{''}})}^{2}}/{(2\sigma_{ji^{'}{vi}^{''}}^{2})}}}{\sqrt{2\pi\sigma_{ji^{'}vi^{''}}^{2}}}$ (8)

This means that for each discrete value of *x_i’_* the continuous feature *x_i’’_* has a separate mean *μ_ji’vi’’_* and standard deviation *σ_ji’vi’’_*.

**THE EXPECTATION MAXIMIZATION ALGORITHM**

Briefly, the Expectation Maximization (EM) method is a generalization of the maximum likelihood estimation of incomplete data,^2^ and is a clustering method suitable for incomplete data, and with different data types such as continuous and discrete features typical of a clinical dataset.

The EM algorithm consists of two steps that are run iteratively until convergence (which we set to when the maximum change of cluster belonging probabilities was less than 10^-6^ between two iterations, or a maximum of 1,000 iterations). In the Expectation step, the probability for each patient of belonging to each cluster is calculated, given the cluster parameters. In the Maximization step, the parameters of each cluster are estimated based on the patients belonging to the cluster. The process is initialized by randomly assigning a subset of the patients to each cluster (in our study, we randomly assigned 100 patients to each cluster).

During the Expectation step, the probability that patient *k* belongs to cluster *c_j_* is calculated, using the above expressions, as:

$P\left( c_{j} \right|\boldsymbol{X}_{k})= \frac{\pi_{j}P\left( \boldsymbol{X}_{k} \right|c_{j})}{\sum_{j} \pi_{j}P\left( \boldsymbol{X}_{k} \right|c_{j^{'}})}$ (9)

During the Maximization step, the parameters of the cluster distributions are estimated. It is known that when using Expectation Maximization in high dimensional spaces and with a large number of clusters, there is a risk of degenerated solutions, either in the sense that some cluster probabilities π_j_ becomes zero, or that the standard deviations of some of the involved distributions becomes zero. To avoid such degenerate solutions, we use Bayesian regularization during estimation. When using conjugate priors, it is possible to think of the prior as being worth a number of observations with some distribution. When estimating each cluster, we use a prior of one sample worth corresponding to the distribution of the whole data set. To this end we first estimate the distribution of each feature from the full data set. Such global estimates have a star superscript in the equations below. α is the strength of the prior, and was in this study set to 1/*C*, where *C* is the number of clusters. *n* is the total number of patients. In detail, the parameters are estimated as:

$p_{iv}^{*}= \frac{\sum_{k} \boldsymbol{1}_{v}(x_{ki})}{n}$ discrete *x_i_* (10)

$\mu_{i}^{*}= \frac{\sum_{k} x_{ki}}{n}$ continuous *x_i_* (11)

$\sigma_{i}^{*}= \frac{\sum_{k} {(x_{ki}- \mu_{i}^{*})}^{2}}{n}$ continuous *x_i_* (12)

$n_{j}= \sum_{k} P\left( c_{j} \right|\boldsymbol{X}_{k})$ (13)

$\pi_{j}= \frac{n_{j}+ \alpha}{n+C\alpha}$ (14)

$p_{jiv= \frac{\sum_{k} \boldsymbol{1}_{v}\left( x_{ki} \right)P\left( c_{j} \right|\boldsymbol{X}_{k})+ \alpha p_{iv}^{*}}{n_{j}+ \alpha}}$ discrete *x_i_* (15)

$p_{ji'i''vw}= \frac{\sum_{k} \boldsymbol{1}_{v}\left( x_{ki^{'}} \right)\boldsymbol{1}_{w}\left( x_{ki^{''}} \right)P\left( c_{j} \right|\boldsymbol{X}_{k})+ \alpha p_{i'v}^{*}p_{i''w}^{*}}{n_{j}+ \alpha}$ discrete *x_i’_* and *x_i’’_* (16)

$\mu_{ji}= \frac{\sum_{k} x_{ki}P\left( c_{j} \right|\boldsymbol{X}_{k})+ \alpha\mu_{i}^{*}}{n_{j}+\alpha}$ continuous *x_i_* (17)

$\sigma_{ji}^{2}= \frac{\sum_{k} {(x_{ki}-\mu_{ji})}^{2}+ \alpha{(\mu_{ji}-\mu_{i}^{*})}^{2}+ \alpha\sigma_{i}^{*2}}{n_{j}+ \alpha}$ continuous *x_i_* (18)

$\boldsymbol{\mu}_{jl}= \frac{\sum_{k} \boldsymbol{x}_{kl}P\left( c_{j} \right|\boldsymbol{X}_{k})+ \alpha\boldsymbol{\mu}_{l}^{*}}{n_{j}+\alpha}$ continuous *x_i_* (19)

$\Sigma_{jl}= \frac{\sum_{k} \left( \boldsymbol{x}_{kl}-\boldsymbol{\mu}_{\boldsymbol{jl}} \right)\left( \boldsymbol{x}_{\boldsymbol{kl}}-\boldsymbol{\mu}_{jl} \right)^{'}P\left( c_{j} \right|\boldsymbol{X}_{k})+ \alpha\left( \boldsymbol{\mu}_{jl}-\boldsymbol{\mu}_{l}^{*} \right)\left( \boldsymbol{\mu}_{jl}-\boldsymbol{\mu}_{l}^{*} \right)^{'}+ \alpha\Sigma_{l}^{*}}{n_{j}+ \alpha}$ continuous *x_i’_* and x_i’’_  (20)

$\mu_{ji^{'}{vi}^{''}}= \frac{\sum_{k} \boldsymbol{1}_{v}(x_{{ki}^{'}})x_{ki^{''}}P\left( c_{j} \right|\boldsymbol{X}_{k})+ \alpha p_{i^{'}v}^{*}\mu_{i^{''}}^{*}}{n_{j}+ \alpha}$ discrete *x_i’_*, continuous *x_i’’_* (21)

$$\sigma_{ji^{'}vi^{''}}^{2}= \frac{\sum_{k} \boldsymbol{1}_{v}\left( x_{ki^{'}} \right){(x_{ki^{''}}-\mu_{ji^{'}vi^{''}})}^{2}P\left( c_{j} \right|\boldsymbol{X}_{k})+ \alpha{(\mu_{ji^{'}vi^{''}}- \mu_{i^{''}}^{*})}^{2}+ \alpha p_{i^{'}v}^{*}\sigma_{i^{''}}^{2}}{n_{j}+ \alpha}$$

discrete *x_i’_*, cont. *x_i’’_* (22)

In the above, ***μ****^*^_l_* is a vector with elements *μ^*^_i’_* and *μ^*^_i’’_*, and Σ*^*^_l_* is a diagonal matrix with diagonal elements σ^2^_i’_ and σ^2^_i’’_. ***1****_v_* is an indicator function which returns 1 if its argument equals *v*, and 0 otherwise.

### **DETERMINATION OF NUMBER OF CLUSTERS**

Specifying the optimal number (*n*) of clusters and ensuring that the algorithm reaches a stable (global) optimum rather than one of several possible local optima is non-trivial and requires careful attention. If the data are well divided in several denser groups, and we try to model it with a mixture model with that same number of clusters, it can be expected to find one solution which is significantly better than other potential solutions, i.e., the one where each denser region is assigned to one cluster. However, if we try to model the same data with a mixture model of too few clusters, some cluster must cover two or more denser regions, and there are many distinct ways to select which regions to agglomerate. Likewise, if we try to model with too many clusters, there are many ways to select which denser regions will be shared between two or more clusters. Thus, if we run the EM algorithm repeatedly, the clustering is expected to be more stable, finding a similar cluster result every time, when the mixture in fact contains several clusters fitting the number of dense regions well. This is the rationale for the used method described here to identify a suitable number of clusters.

One way to alleviate the issue with local maxima is to run the algorithm multiple times with random starting points, choosing the model with the highest log likelihood. The same approach of running the EM algorithm several times can also be used to assess the stability of the clustering and be used to decide on a suitable number of clusters.

A traditional way of deciding on an optimal number of clusters is to compare the log-likelihood between models with different number of clusters. However, the log-likelihood tends to increase with the number of clusters and is therefore not an unambiguous indicator for the best number of clusters.

To find an optimal number of clusters for our model, ten different models of three to fifteen clusters were created. The log likelihood for each model was calculated, and for each cluster number the model with the highest log likelihood was selected. This process was repeated twenty times, to make twenty different models, each with the highest log likelihood in a set of ten models. To focus on three to fifteen clusters was a clinical trade off – too many clusters might not be clinically relevant, despite the risk that they may represent potentially important separation of phenotypes. However, within this range a methodological optimum may be identified.

For the twenty selected models, each with highest log likelihood in a set of ten models, the cluster similarity was determined by using a cluster similarity index (CSI) defined as the fraction of patients who were assigned to the same cluster in model a as in model b.^3^ As the starting points for the clustering were randomly assigned, cluster labels could differ between models. Relabelling of the clusters was required to make comparison possible between models. This was done by finding the permutation of clusters between two models which gave the highest match between cluster indices between the patients in the two models.

CSI was calculated between all pairs of the models. Median and inter-quartile range (IQR) was calculated for each number of clusters. As the CSI, when numbers of clusters << number of patients, by nature is higher for lower number of clusters, a penalty for the number of clusters was added by subtracting 1/*n* clusters from all median CSI. The number of clusters with the highest CSI was determined to be optimal, as this indicates that the clustering was most stable with this number of clusters. When describing model parameters, the model of the optimal number of clusters with the highest log likelihood was chosen to represent our model.

**REFERENCES**

1 Chow C, Liu C. Approximating discrete probability distributions with dependence trees. *IEEE Transactions on Information Theory* 1968; **14**: 462–7.

2 Do CB, Batzoglou S. What is the expectation maximization algorithm? *Nature Biotechnology* 2008; **26**: 897–9.

3 Lange T, Roth V, Braun ML, Buhmann JM. Stability-based validation of clustering solutions. *Neural Computation* 2004; **16**: 1299–323.
