# Supplementary material for: Clustering identifies endotypes of traumatic brain injury in an intensive care cohort: a CENTER-TBI study
Source: Crit Care. 2022 Jul 27;26:228. doi: 10.1186/s13054-022-04079-w (PMC9327174; doi:10.1186/s13054-022-04079-w)
Supplement: Supplementary file 4 — Additional file 4: Table of Cluster means and overall means, min, max and standard deviation of all features. [file 13054_2022_4079_MOESM4_ESM.docx]

| Table 1: Cluster means and overall means, min, max and standard deviation of all features. | | | | | | | | | |
| --- | --- | --- | --- | --- | --- | --- | --- | --- | --- |
| **Cluster** | ***All patients*** | **A** | **B** | **C** | **D** | **E** | **F** | **MI** | |
| N patients | *1728* | 497 | 262 | 48 | 343 | 360 | 218 |  | |
|  |  |  |  |  |  |  |  |  | |
| **ASA-PS classification** |  |  |  |  |  |  |  | 0.010 | |
| 1 | *922 (55.9)* | 275 (57.2) | 112 (45.2) | 23 (49) | 182 (55.5) | 203 (60.8) | 127 (59.9) |  | |
| 2 | *545 (33)* | 153 (31.8) | 97 (39.1) | 12 (25.6) | 120 (36.6) | 102 (30.5) | 61 (28.8) |  | |
| 3 | *170 (10.3)* | 47 (9.8) | 36 (14.5) | 11 (23.4) | 25 (7.6) | 28 (8.4) | 23 (10.8) |  | |
| 4 | *13 (0.8)* | 6 (1.2) | 3 (1.2) | 1 (2.1) | 1 (0.3) | 1 (0.3) | 1 (0.5) |  | |
|  |  |  |  |  |  |  |  |  | |
| Anticoagulant or anti-platelet treatment pre-injury | *282*  *(17.3)* | 95 (19.9) | 49 (20.2) | 11 (25.6) | 55 (16.9) | 40 (12) | 32 (15.7) | 0.004 | |
| BMI [kg/m^2^] at arrival | *24.8*  *(22.9-27.7)* | 25.4  (23.0-27.8) | 25.78  (23.3-27.8) | 25.15  (23.1-27.0) | 24.5  (22.4-26.7) | 24.7  (22.9-27.6) | 25.1  (23.2-27.8) | 0.020 | |
|  |  |  |  |  |  |  |  |  | |
| **Type of injury** |  |  |  |  |  |  |  | 0.017 | |
| Closed | *1575 (92.8)* | 459 (94.6) | 242 (93.4) | 47 (97.9) | 325 (97) | 313 (88.4) | 189 (87.1) |  | |
| Blast | *2 (0.1)* | 1 (0.2) | (0) | (0) | (0) | (0) | 1 (0.5) |  | |
| Crush | *45 (2.7)* | 8 (1.6) | 4 (1.5) | (0) | 3 (0.9) | 21 (5.9) | 9 (4.1) |  | |
| Penetrating | *14 (0.8)* | 4 (0.8) | 3 (1.2) | 1 (2.1) | (0) | 4 (1.1) | 2 (0.9) |  | |
| Penetrating-Perforating | *8 (0.5)* | 1 (0.2) | 1 (0.4) | (0) | 2 (0.6) | 2 (0.6) | 2 (0.9) |  | |
| Penetrating-tangential | *1 (0.1)* | (0) | 1 (0.4) | (0) | (0) | (0) | (0) |  | |
| Closed w open  depressed skull fracture | *53 (3.1)* | 12 (2.5) | 8 (3.1) | (0) | 5 (1.5) | 14 (4) | 14 (6.4) |  | |
|  |  |  |  |  |  |  |  |  | |
| **Cause of injury** |  |  |  |  |  |  |  | 0.031 | |
| RTC | *745 (46.5)* | 198 (42.7) | 77 (31.4) | 11 (26.9) | 177 (55.8) | 172 (52.3) | 110 (53.7) |  | |
| Incidental fall | *700 (43.7)* | 221 (47.6) | 148 (60.4) | 26 (63.3) | 118 (37.2) | 119 (36.2) | 68 (33.2) |  | |
| Other non-  intentional injury | *58 (3.6)* | 15 (3.2) | 9 (3.7) | 2 (4.9) | 11 (3.5) | 14 (4.3) | 7 (3.4) |  | |
| Violence/Assault | *61 (3.8)* | 22 (4.7) | 10 (4.1) | 2 (4.9) | 10 (3.2) | 9 (2.7) | 8 (3.9) |  | |
| Act of mass violence | *1 (0.1)* | 1 (0.2) | (0) | (0) | (0) | (0) | (0) |  | |
| Suicide attempt | *36 (2.2)* | 7 (1.5) | 1 (0.4) | (0) | 1 (0.3) | 15 (4.6) | 12 (5.9) |  | |
|  |  |  |  |  |  |  |  |  | |
| **Pupillary reactivity** |  |  |  |  |  |  |  | 0.068 | |
| Both reacting | *1308 (80.1)* | 450 (95.9) | 193 (78.1) | 28 (70) | 280 (85.6) | 219 (62.9) | 138 (68.3) |  | |
| One reacting | *114 (7)* | 18 (3.8) | 19 (7.7) | 2 (5) | 21 (6.4) | 38 (10.9) | 16 (7.9) |  | |
| Both unreactive | *211 (12.9)* | 1 (0.2) | 35 (14.2) | 10 (24.9) | 26 (8) | 91 (26.1) | 48 (23.8) |  | |
|  |  |  |  |  |  |  |  |  | |
| GCS Motor Score | *5 (1-6)* | 6 (6-6) | 5 (2.5-5) | 5 (3.5-5) | 4 (2-5) | 1 (1-1) | 4 (1-5) | 1.436 | |
| GCS Total Score | *9 (4-14)* | 15 (14-15) | 9 (6-12) | 9 (6.75-13) | 7.5 (6-10) | 3 (3-3) | 7 (4-10) | 1.295 | |
| Hypoxic event before ICU admission, definite or suspect | *232 (13.6)* | 17 (3.5) | 22 (8.5) | 4 (8.4) | 28 (8.2) | 85 (23.9) | 76 (35.2) | 0.049 | |
| Hypotensive event before ICU admission, definite or suspect | *239 (14.0)* | 29 (6) | 18 (7) | 8 (16.7) | 26 (7.6) | 87 (24.4) | 71 (32.9) | 0.040 | |
| MAP [mmHg] | *97*  *(85-110)* | 98  (87-110) | 103  (93-117) | 94  (87-103) | 98  (87-112) | 94  (80-108) | 90  (73-107) | 0.049 | |
| Heart Rate [min^-1^] | *82*  *(71-99)* | 80  (70-92) | 80  (70-91) | 85  (70-103) | 80  (70-92) | 85  (72-101) | 95  (80-119) | 0.056 | |
| Body Temperature [°C] | *36*  *(35.4-36.7)* | 36.5  (35.9-36.9) | 36.2  (35.5-36.7) | 35.7  (34.3-36.6) | 36  (35.4-36.6) | 35.9  (35-36.6) | 35.8  (34.8-36.4) | 0.118 | |
| SpO_2_ [%] | *99 (96-100)* | 98 (96-100) | 98 (96-100) | 98 (95-100) | 100 (99-100) | 99 (97-100) | 95 (85-98) | 0.692 | |
|  |  |  |  |  |  |  |  |  | |
| **Labs** |  |  |  |  |  |  |  |  | |
| pH | *7.35*  *(7.28-7.39)* | 7.37  (7.32-7.41) | 7.35  (7.31-7.4) | 7.27  (7.085-7.4) | 7.36  (7.32-7.39) | 7.32  (7.2475-7.39) | 7.29  (7.2-7.36) | 0.228 | |
| Base Excess | *-2.9*  *(-5.7- -0.9)* | -1.7  (-3.725-0.2) | -3.15  (-5.3- -1.1) | -3.9  (-12.1-0.6) | -2.3  (-4- -1) | -3.6  (-6.6- -1) | -5  (-7.9- -2) | 0.234 | |
| PaO_2_ [kPa] | *20.4*  *(11.6-35.2)* | 17.9  (11.0-27.9) | 16.5  (10.4-26.0) | 22.5  (12.4-40.8) | 26.3  (15.9-39.5) | 23.6  (12.7-41.1) | 16.9  (10.4-30.7) | 0.067 | |
| PaCO_2_ [kPa] | *5.5 (4.8-6.2)* | 5.3 (4.8-6.0) | 5.3 (4.7-6.0) | 5.3 (4.4-5.8) | 5.4 (5.0-6.0) | 5.6 (4.8-6.7) | 5.9 (5.0-7.2) | 0.141 | |
| Lactate [mmol/L] | *2.2 (1.4-3.4)* | 2.0 (1.2-2.7) | 2.3 (1.4-3.4) | 4.9 (2.2-8.1) | 1.7 (1.2-2.4) | 2.2 (1.4-3.4) | 5.3 (2.9-10.0) | 0.884 | |
| Glucose [mmol/L] | *7.7 (6.5-9.4)* | 7.2 (6.3-8.4) | 8.0 (6.7-9.3) | 8.5 (6.9-14.3) | 7.3 (6.3-8.6) | 8.1 (6.8-10.5) | 9.1 (6.9-11.8) | 0.255 | |
| Sodium [mmol/L] | *140*  *(138-142)* | 140  (138-142) | 140  (138-143) | 140  (136-143) | 140  (138-142) | 141  (138-143) | 140  (138-143) | 0.057 | |
| Platelet count [*10^9^/L] | *208*  *(165-250)* | 217  (174-256) | 202  (166-258) | 184  (132-236) | 206  (167-244) | 195  (159-250) | 209  (158-245) | 0.012 | |
| Creatinine [µmol/L] | *75 (62-89)* | 76 (64-88) | 70 (58-86) | 106 (64-257) | 71 (60-83) | 74 (59-91) | 83 (71-101) | 0.630 | |
| Haemoglobin [g/L] | *132 (116-144)* | 138 (123-148) | 132 (120-143) | 119 (100-134) | 130 (113-144) | 128 (111-140) | 129 (112-142) | 0.024 | |
| **CT Characteristics** |  |  |  |  |  |  |  |  | |
| Rotterdam CT Score | *3 (3-4)* | 3 (2-3) | 4 (3-5) | 3 (2-5) | 3 (3-4) | 3 (3-5) | 3 (3-4) | 0.072 | |
| 1 | *5 (0.3)* | 3 (0.7) | 0 (0) | 1 (2.3) | 1 (0.3) | 0 (0) | 0 (0) |  | |
| 2 | *312 (20.2)* | 153 (33.6) | 16 (7.1) | 11 (25.6) | 43 (14.6) | 41 (12.6) | 48 (24.2) |  | |
| 3 | *699 (45.4)* | 218 (47.9) | 89 (39.6) | 17 (39.6) | 160 (54.4) | 123 (37.7) | 92 (46.5) |  | |
| 4 | *213 (13.8)* | 46 (10.1) | 62 (27.5) | 2 (4.7) | 28 (9.5) | 51 (15.6) | 24 (12.1) |  | |
| 5 | *219 (14.2)* | 28 (6.2) | 42 (18.7) | 7 (16.3) | 50 (17) | 71 (21.8) | 21 (10.6) |  | |
| 6 | *93 (6)* | 7 (1.5) | 16 (7.1) | 5 (11.6) | 12 (4.1) | 40 (12.3) | 13 (6.6) |  | |
| Fisher Classification | *2 (2-3)* | 2 (1-2) | 2 (2-4) | 2 (1.5-3.5) | 2 (2-4) | 2 (2-4) | 2 (2-3) | 0.047 | |
| No tSAH, no IVH | *342 (22.2)* | 168 (36.9) | 19 (8.5) | 11 (25.6) | 55 (18.8) | 41 (12.6) | 48 (24.2) |  | |
| No IVH, trace or moderate tSAH | *703 (45.6)* | 205 (45.1) | 114 (50.7) | 17 (39.6) | 144 (49.1) | 135 (41.4) | 88 (44.4) |  | |
| No IVH, full tSAH | *124 (8.1)* | 23 (5.1) | 28 (12.4) | 4 (9.3) | 19 (6.5) | 37 (11.3) | 13 (6.6) |  | |
| IVH | *371 (24.1)* | 59 (13) | 64 (28.4) | 11 (25.6) | 75 (25.6) | 113 (34.7) | 49 (24.7) |  | |
| Midline Shift (mm) | *0 (0-2)* | 0 (0-0) | 2 (0-5) | 0 (0-4) | 0 (0-0) | 0 (0-5) | 0 (0-0) | 0.077 | |
| TAI | *239 (15.7)* | 34 (7.5) | 23 (10.6) | 2 (4.9) | 67 (22.8) | 69 (21.6) | 44 (22.3) | 0.020 | |
| EDH | *280 (18.4)* | 76 (16.8) | 58 (26.6) | 6 (14.6) | 50 (17) | 57 (17.8) | 33 (16.9) | 0.003 | |
| aSDH | *737 (48.5)* | 150 (33.3) | 150 (68.8) | 24 (58.5) | 146 (49.8) | 178 (55.6) | 89 (45.2) | 0.029 | |
| Contusion | *855 (56.2)* | 205 (45.4) | 175 (80.6) | 17 (41.5) | 190 (64.6) | 187 (58.4) | 81 (41.1) | 0.038 | |
| Decompressive Craniectomy | *216 (12.5)* | 22 (4.4) | 48 (18.3) | 7 (14.6) | 40 (11.7) | 73 (20.3) | 26 (11.9) | 0.08 | |
|  |  |  |  |  |  |  |  |  | |
| Head ISS | *25 (16-25)* | 16 (9-16) | 25 (16-25) | 25 (16-25) | 25 (16-25) | 25 (25-25) | 25 (16-25) | 0.159 | |
| Highest Extracranial ISS | *9 (0-16)* | 4 (0-16) | 4 (0-9) | 9 (0-10.75) | 9 (0-16) | 9 (0-16) | 9 (0.25-16) | 0.035 | |
| Intubation | *1334 (78.9)* | 218 (45.7) | 224 (85.8) | 39 (84.8) | 314 (93.2) | 347 (97.5) | 192 (89.7) |  | |
| ICP monitoring | *757 (44.2)* | 76 (15.6) | 153 (58.4) | 19 (40.4) | 177 (51.8) | 225 (62.7) | 107 (49.5) |  | |
| Median Daily TIL | *2 (0-5.5)* | 0.5 (0-1.5) | 3.5 (0.5-8) | 2 (0-3.25) | 3 (1-5) | 4.5 (2-8.5) | 3 (1-5.625) |  | |
| **GOS-E 6 months**  **post-injury** |  |  |  |  |  |  |  | 0.083 | |
| 1 | *388 (22.5)* | 34 (6.8) | 77 (29.4) | 19 (39.6) | 62 (18.1) | 135 (37.5) | 61 (28) |  | |
| 2 or 3 | *268 (15.5)* | 40 (8) | 50 (19.1) | 5 (10.4) | 70 (20.4) | 68 (18.9) | 35 (16.1) |  | |
| 4 | *123 (7.1)* | 31 (6.2) | 19 (7.3) | 6 (12.5) | 27 (7.9) | 21 (5.8) | 19 (8.7) |  | |
| 5 | *241 (13.9)* | 71 (14.3) | 34 (13) | 5 (10.4) | 49 (14.3) | 47 (13.1) | 35 (16.1) |  | |
| 6 | *214 (12.4)* | 70 (14.1) | 31 (11.8) | 5 (10.4) | 51 (14.9) | 29 (8.1) | 28 (12.8) |  | |
| 7 | *229 (13.3)* | 107 (21.5) | 24 (9.2) | 2 (4.2) | 40 (11.7) | 37 (10.3) | 19 (8.7) |  | |
| 8 | *265 (15.3)* | 144 (29) | 27 (10.3) | 6 (12.5) | 44 (12.8) | 23 (6.4) | 21 (9.6) |  | |
| GOS-E and Decompressive craniectomy was not used for clustering but is shown here for reference. BMI, body mass index; RTC, road traffic collision; GCS, Glasgow coma scale; ICU, intensive care unit; MAP, mean arterial pressure; SpO_2_, oxygen saturation; PaO_2_, arterial partial pressure of oxygen; PaCO_2_, arterial partial pressure of carbon dioxide; tSAH, traumatic subarachnoid haemorrhage; IVH, intraventricular haemorrhage; TAI, traumatic axonal injury; EDH, epidural hematoma; aSDH, acute subdural hematoma; ISS, injury severity score; ICP, intracranial pressure; TIL, therapy intensity level; GOS-E, Glasgow outcome scale extended. | | | | | | | | | |
